# Severe pulmonary embolism in COVID-19 patients: a call for increased awareness

**DOI:** 10.1186/s13054-020-02931-5

**Published:** 2020-06-02

**Authors:** Guillaume Hékimian, Guillaume Lebreton, Nicolas Bréchot, Charles-Edouard Luyt, Matthieu Schmidt, Alain Combes

**Affiliations:** 1 Sorbonne Université, INSERM, UMRS_1166-ICAN, Institute of Cardiometabolism and Nutrition, F-75013 Paris, France; 2 Service de médecine intensive-réanimation, Institut de Cardiologie, APHP Hôpital Pitié–Salpêtrière, F-75013 Paris, France; 3 Service de chirurgie thoracique et cardio-vasculaire, Institut de Cardiologie, APHP Hôpital Pitié–Salpêtrière, F-75013 Paris, France

Dear Editor,

Coronavirus disease 2019 (COVID-19) is associated with severe systemic inflammation and important elevation of fibrinogen and D-dimers that has been associated with a poor prognosis [1, 2]. This proinflammatory state might favor severe thromboembolic events and pulmonary embolism (PE).

We retrospectively reviewed characteristics of patients with confirmed SARS-CoV-2 infection and acute PE who were admitted to our tertiary ICU, which serves as an ECMO referral center for the Greater Paris. In accordance with the ethical standards of French legislation, only non-opposition of patient’s surrogate for utilization of the deidentified data was obtained. The ICU database was registered with the national data protection authority (CNIL 1950673). No analysis for statistical significance was performed given the descriptive nature of the study.

From February 25, 2020, to April 6, 2020, 51 patients with confirmed SARS-CoV-2 infection were treated in our ICU, of whom 8 (16%) had confirmed severe PE. Patients’ main characteristics are described in Table 1. Four patients had PE while on VV-ECMO for severe ARDS. VA-ECMO was initiated in 3 other patients with refractory shock due to right ventricular failure, and one patient died of refractory cardiac arrest before ECMO could be installed. PE was suspected in 6 patients because of acute cor pulmonale at echocardiographic evaluation (online supplementary data). PE diagnosis was confirmed by CT angiography (online supplementary data) in 7 patients and by autopsy in one patient. All except one had received anticoagulation before PE diagnosis. Five patients had a very high level of fibrinogen, and all had important increase in D-dimers. As of April 6, 2020, 3 patients had died of multiple organ failure and 5 are still on MV and ECMO in the ICU.

**Table 1:** Characteristics of the 8 COVID-19 patients who developed massive pulmonary embolism

	Patient 1	Patient 2	Patient 3	Patient 4	Patient 5	Patient 6	Patient 7	Patient 8
Baseline characteristics								
Age	60	64	41	59	65	61	55	49
Gender	M	M	M	F	M	F	F	F
BMI, kg/m^2^	32	27	31	39	32	27	33	30
Comorbidities								
Hypertension	0	1	1	1	1	1	0	1
Diabetes	1	0	0	0	0	0	0	0
Active smoker	0	0	0	0	0	0	0	0
Heart disease	1	0	0	0	1	0	0	0
Stroke	0	1	0	0	0	0	0	0
Clinical characteristics at the time of PE diagnosis								
MV duration, days	7	8	3	10	0	4	9	1
Reason for pulmonary embolism suspicion*	1, 2, 3	1, 3	1, 2	1	4	2, 3	1, 2, 3	1
Norepinephrine, mg/h	50	3	0	0	0	0.6	0	5
Acute kidney injury requiring dialysis	0	1	0	1	0	0	0	0
On VV-ECMO for severe ARDS	0	0	1	1	0	1	1	0
Anticoagulant therapy	Dalteparin, 5000 U/day	UFH, 12,000 U/day	Enoxaparin, 6000 U/day	UFH, 12,000 U/day	None	Enoxaparin, 6000 U/day	Enoxaparin, 8000 U/day	Enoxaparin, 8000 U/day
Laboratory findings at the time of PE diagnosis								
D-dimers, ng/mL	–	7280	16,450	> 20,000	–	15,360	> 20,000	> 20,000
Fibrinogen, g/dl	8.7	7.8	9.8	3.6	3.2	8.2	6.3	2.5
aPTT ratio**	1.05	1.18	1.27	1.14	–	–	1.23	1.2
Anti-Xa activity, U/mL	–	< 0.2	–	< 0.2	–	–	< 0.2	< 0.2
Troponin, ng/L	< 13	28	131	59	13	16	42	168
White blood cell count, G/L	13.8	16.1	16.5	23.4	6.1	11	17.5	12.8
Neutrophils, g/dl	13	14.4	14.2	20.6	3.4	9.9	15.3	10.9
Lymphocytes, g/dl	0.4	0.5	0.7	1.7	1.7	0.7	1.3	1.3
Hemoglobin, g/dl	13	11.5	12.3	10.3	11.7	7.4	7	10.5
Platelet count, × 10^3^/μL	244	610	242	128	114	237	335	228
pH	7.06	6.94	7.41	7.45	7.27	7.29	7.2	7.06
pCO_2_, mmHg	63	59	41	40	43	70	79	36
pO2, mmHg	40	75	54	66	84	60	60	177
PO_2_/FiO_2_	40	75	54	69	NA	66	60	177
Lactate, mmol/L	28	9	2	1.7	1.4	0.9	1.7	8.5
ASAT, U/L	37	43	108	71	53	63	62	2509
ALAT, U/L	23	29	45	67	24	42	96	1207
LDH, U/L	–	512	821	894	264	388	541	5884
Total bilirubin, mmol/L	8	61	12	16	22	41	8	25
Creatinine kinase, IU/L	34	103	3648	105	41	234	203	151
Outcomes								
Received VA-ECMO for PE-associated shock	1	1	0	0	0	0	0	1
Current status as of April 6, 2020	Dead	Dead	In ICU, ongoing MV and ECMO	In ICU, ongoing MV and ECMO	Dead	In ICU, ongoing MV and ECMO	In ICU, ongoing MV and ECMO	In ICU, ongoing MV and ECMO

*BMI* body mass index, *PE* pulmonary embolism, *MV* mechanical ventilation, *VA-ECMO* veno-arterial extracorporeal membrane oxygenation, *VV-ECMO* veno-venous extracorporeal membrane oxygenation, *aPTT* activated partial thromboplastin time

*Reason for pulmonary embolism suspicion: 1 = acute cor pulmonale on Doppler echocardiography, 2 = worsening hypoxemia, 3 = hypercapnia with preserved respiratory system compliance, and 4 = fortuitous discovery

**Ratio of the patient aPPT to the control aPPT used by the laboratory (control aPPT = 33 s for the La Pitié Salpêtrière laboratory)

We describe a series of 8 critically ill patients with massive PE following COVID-19 infection. Four of these patients developed PE while on VV-ECMO for severe ARDS, a condition that was not reported in the 156 patients included in the EOLIA trial [3] who received ECMO and in the 350 VV-ECMO patients of the LIFEGARDS international multicenter prospective cohort [4]. Interestingly, 7 of the 8 patients had received preventive anticoagulation that did not prevent PE. In 6 of the 8 patients, Doppler echocardiography showing acute right ventricle dilation prompted CT angiography that confirmed PE.

Massive PE in COVID-19 patients may be the consequence of sepsis-induced disseminated intravascular coagulation or to a specific procoagulant state caused by inflammation or virus-induced endothelial dysfunction [5]. Important elevation of D-dimers was indeed reported in these patients and was associated with subsequent ARDS and in-hospital mortality [5]. However, massive PE was not reported in previous series [1, 2], although it may have been the unproven cause of death in some patients.

Our observation has potential major clinical implications. First, higher level of anticoagulation might be considered in patients with the most severe forms of the disease, those with high D-dimers and, contrarily to our previous recommendation, in patients supported by VV-ECMO [3]. Second, routine Doppler echocardiography should be performed daily to detect early signs of acute cor pulmonale in critically ill patients. Lastly, PE should also be suspected in COVID-19 patients with worsening hypoxemia or hypercapnia under mechanical ventilation.

This case series has several limitations. It is a small single-center case series of critically ill patients, we did not compare clinical and biological characteristics of patients with or without PE, and PE incidence could not be accurately estimated. However, we think that physicians should be warned about the occurrence of severe and potentially fatal PE in COVID-19 patients.

## Supplementary information

**Additional file 1:**

## Data Availability

The datasets generated during the current study are available from the corresponding author on reasonable request.
